# Why don't we share data and code? Perceived barriers and benefits to public archiving practices

**DOI:** 10.1098/rspb.2022.1113

**Published:** 2022-11-30

**Authors:** Dylan G. E. Gomes, Patrice Pottier, Robert Crystal-Ornelas, Emma J. Hudgins, Vivienne Foroughirad, Luna L. Sánchez-Reyes, Rachel Turba, Paula Andrea Martinez, David Moreau, Michael G. Bertram, Cooper A. Smout, Kaitlyn M. Gaynor

**Affiliations:** ^1^ NRC Research Associate, Northwest Fisheries Science Center, National Marine Fisheries Service, National Oceanic and Atmospheric Administration, Seattle, WA 98112, USA; ^2^ Cooperative Institute for Marine Resources Studies, Hatfield Marine Science Center, Oregon State University, Newport, OR 97365, USA; ^3^ Evolution & Ecology Research Centre, School of Biological, Earth and Environmental Sciences, The University of New South Wales, Sydney, New South Wales 2052, Australia; ^4^ Earth and Environmental Sciences Area, Lawrence Berkeley National Laboratory, Berkeley, CA 94720, USA; ^5^ Department of Biology, Carleton University, Ottawa, Canada, K1S 5B6; ^6^ Department of Biology, Georgetown University, Washington, DC 20057, USA; ^7^ School of Natural Sciences, University of California, Merced, 95343 USA; ^8^ Department of Ecology and Evolutionary Biology, University of California, Los Angeles, CA 90095-7239, USA; ^9^ Australian Research Data Commons, The University of Queensland, Brisbane 4072, Australia; ^10^ School of Psychology and Centre for Brain Research, University of Auckland, Auckland 1010, New Zealand; ^11^ Department of Wildlife, Fish, and Environmental Studies, Swedish University of Agricultural Sciences, Umeå, SE-907 36, Sweden; ^12^ Institute for Globally Distributed Open Research and Education (IGDORE), Brisbane 4001, Australia; ^13^ Departments of Zoology and Botany, University of British Columbia, Vancouver, Canada, BC V6T 1Z4; ^14^ National Center for Ecological Analysis and Synthesis, Santa Barbara, CA 93101, USA

**Keywords:** open science, data science, reproducibility, transparency, data reuse, code reuse‌

## Abstract

The biological sciences community is increasingly recognizing the value of open, reproducible and transparent research practices for science and society at large. Despite this recognition, many researchers fail to share their data and code publicly. This pattern may arise from knowledge barriers about how to archive data and code, concerns about its reuse, and misaligned career incentives. Here, we define, categorize and discuss barriers to data and code sharing that are relevant to many research fields. We explore how real and perceived barriers might be overcome or reframed in the light of the benefits relative to costs. By elucidating these barriers and the contexts in which they arise, we can take steps to mitigate them and align our actions with the goals of open science, both as individual scientists and as a scientific community.

## 1. Introduction

Science is an iterative process in which our understanding of the world is continually updated with new information. Open, reproducible and transparent science practices allow us to more quickly and reliably evaluate, replicate and integrate studies to advance our knowledge [1–3]. A key component of open science is the publishing of datasets and analytical code used to make scientific inference [4,5]. Given the rapid growth of computational resources to store, process and analyse big data, sharing data and code with the public is easier and more important than ever.

Data and code sharing allows innovative reanalysis with new, improved methods or synthesis with other datasets, potentially leading to new insights [6–8]. Datasets collected for the purpose of answering one particular question can also be valuable assets to future researchers with entirely different questions and goals [9]. As computer programming becomes more necessary and accessible for reproducible data cleaning, processing, model building and statistical analyses, the value of code (e.g. programming scripts or other scientific software) for the scientific community is increasing [5]. While common language can only approximate a useful description of an analytical method, code can guide precise reproduction of the methodology in a research article. Code sharing saves researchers time from ‘reinventing the wheel’ in future projects and allows others to modify existing code for their own purposes.

In addition to advancing the scientific enterprise, publicly sharing data and code can benefit society at large [10–12]. For example, open science practices led to rapid advancements in our understanding of, and thus ability to combat, the emergence of SARS-CoV-2 [13–16]. However, one does not have to be an altruist to share data and code, as there are also many individual benefits that often outweigh any perceived costs [17]. For example, researchers who practice open science benefit from increased citation rates, visibility, collaboration efficiency and ease of future work [18–20].

**Figure 1.**
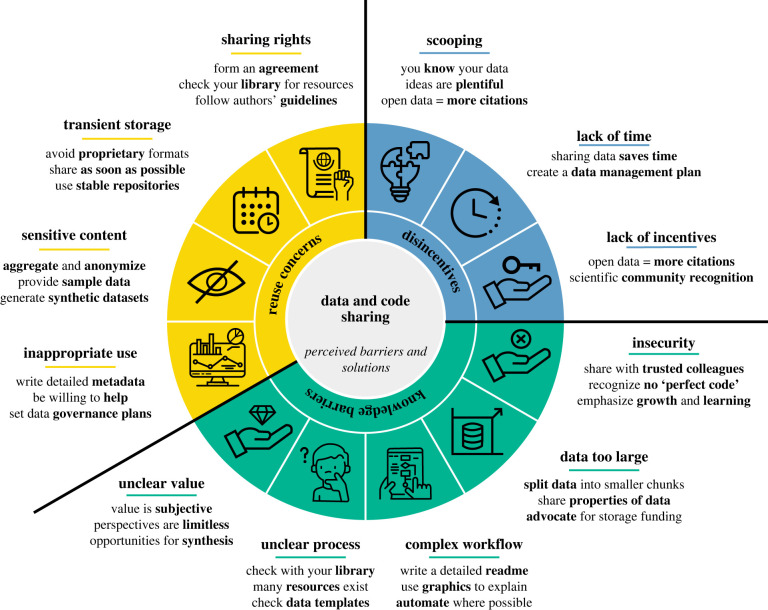
Perceived barriers and solutions to sharing data and code. We highlight 12 distinct barriers (see icons and corresponding underlined titles) to researchers publicly sharing data and code, which can be broken into three larger groups (knowledge barriers, reuse concerns and disincentives; innermost circle). Underneath the section titles, we list a few suggestions for overcoming these barriers (see main text for more details). (Online version in colour.)

Despite the benefits of open science for individual researchers, science and society, many biologists do not publicly share their data and code. We convened a working group at the inaugural (2021) meeting of the Society for Open, Reproducible, and Transparent Ecology and Evolution (SORTEE) to explore barriers to data and code sharing, and this paper is the distillation of our discussion (see description of process in §6 below). Here, we review common reasons for the failure to adopt open data and code practices. We have grouped these reasons into three broad categories: knowledge barriers, reuse concerns and career incentives (figure 1). Our target audience is the individual researcher who is looking to navigate the open science landscape amidst uncertainty and hesitation, and we therefore focus on changes in individual behaviour and offer counterpoints to alleviate the individual researcher's concerns. That said, we recognize the importance of top–down as well as bottom–up change, and we discuss the critical role of journals, funding agencies and research institutions in setting policies to incentivize individual behaviour. We hope that our recommendations empower individuals to both alter their own behaviour and advocate for top–down change, and we encourage readers in decision-making positions to promote systemic change toward more open biological research.

## 2. Knowledge barriers

### (a) Unsure about the process

Many researchers do not share their data and code simply because they do not know how. The process of archiving data and code is not always straightforward. In one survey of biologists, 46% of respondents were unaware of how to organize data in a presentable and usable way, and 33% reported that not knowing which online hosting service, or repository, to use was a barrier to sharing data and code [21]. The choice of repository can depend on multiple factors, like the type of digital output, science domain, size, national policy, funding agency and access restrictions [22–24], although there are general repositories that capture many forms of digital outputs, such as Zenodo (https://www.zenodo.org), OSF (https://osf.io/), Dryad (https://www.datadryad.org) and Figshare (https://figshare.com).

While data and code sharing can be daunting, there is a growing number of online resources and tips to support individuals and teams of researchers through the process (see [25–28]). For example, editorial support staff and institutional libraries often provide free, but under-used, guidance or assistance in archiving data and code [29]. There are also many data and metadata templates to help standardize data and to ensure that data are reusable by others [30–32]. FAIR principles and practices (**F**indability, **A**ccessibility, **I**nteroperability and **R**euse), for example, provide a framework and a set of guidelines that help researchers understand how to share data and code most effectively [33] (https://www.go-fair.org/fair-principles/). These templates will likely save researchers time in the long term, as data will be more organized and readily usable for their own future work [34]. Ultimately, even if data are shared in a repository that may not be the ideal fit, or if the code is not optimized or fully annotated, some form of data and code sharing is better than none. One of the best ways to gain knowledge about data and code sharing is through experience, so we encourage researchers to use the resources that are available to them, not to shy away from publications that require sharing, and accept that their practices will improve over time.

### (b) Complex or manual workflows

When many manual steps are involved in a data workflow, researchers may be unsure about how to share their process in a fully reproducible manner. While the best practice for open science is to process and clean data with reproducible code, researchers have different levels of comfort with programming and some workflows may require manual steps or proprietary software. As a result, some intermediate data products may not be derivable from code alone.

To facilitate reproducibility in these cases, researchers should detail any manual data processing steps or point-and-click selection tools of a workflow in a metadata or readme file that accompanies data and code [35]. These manual workflows include manually summarizing or cleaning data in spreadsheets, cursor-based polygon selections in GIS software or cursor-based acoustic analyses, for example. The manual steps required in between scripts should be described as clearly as possible, and unless the process is highly subjective, the results should be approximately reproducible with sufficient detail. Using non-proprietary documents (e.g. pdf) with embedded images (e.g. screenshots), workflow diagrams, graphical readme files and other explanatory figures as supplemental information to a manuscript can aid the reader in understanding such complex manual steps. It is easy to forget exact steps after data are analysed, thus it is imperative that researchers document these manual steps as carefully as possible throughout the work, starting at the conception of the project.

Of course, seeking ways to reduce manual steps through automation can make for more efficient and reproducible workflows. For example, tools like OpenRefine (https://openrefine.org) can help write ‘recipes’ from point-and-click data cleaning workflows. Manual tasks can be converted to a coding script retroactively, giving downstream users control over the inputs needed in that stage of the workflow. For example, manual point-and-click selection of polygons in GIS software can be turned into code by defining the selected values (e.g. latitude and longitude) after the process. As another example, cursor-based trimming of acoustic files can be turned into a programmatic command that reads a set of input values (e.g. start time and end time) and carries out a function at those inputs (such as with ffmpeg [36]). While the inputs may have been derived manually, the process can be documented in functional steps with code.

### (c) Large data files

Datasets are rapidly growing in size, complexity and quantity, thereby creating logistical barriers to sharing [37,38]. For example, some types of data like remotely sensed satellite imagery or climate model projections can produce terabytes or even petabytes of data per day [38,39]. Even transferring and storing datasets on the order of 1 GB can create challenges arising from file size [40]. Researchers may be wary of the storage space required to publicly share large data files, or unsure of best practices for bundling data into smaller subsets.

As cloud storage capacity grows each year, there are many opportunities for free storage of large research datasets. For example, there is no storage limit at the OSF repository and a 50 GB limit per dataset at Zenodo [22]. In rare cases in which datasets exceed these limitations, dataset managers can bundle smaller datasets for easier upload and downstream data reuse [41]. For example, The Climate Modelling Intercomparison Project (CMIP6) data and associated model runs contain petabytes of data, but are divided into smaller ‘file sets’ for more efficient storage and download [42]. The CMIP6 creators use consistent data and metadata standards [43,44] to ensure that all file sets are interoperable. Even working with smaller observational, experimental or modelling datasets, this process of bundling data files can make data management easier and more organized. Researchers might consider archiving model input and testing data by a relevant subgroup (e.g. by time period, variable groupings, or spatio-temporal resolution) [41]. As ‘big data’ continues to grow, funders, journals and research institutions need to offer financial and personnel support for the storage and maintenance of such large datasets.

### (d) Insecurity

Insecurity, embarrassment and fear can be powerful emotional barriers to publicly sharing data and code. Publicly exposing the behind-the-scenes details of data management and analysis can feel vulnerable, especially for early-career researchers and novice coders [45]. Some may fear a scenario in which others find inaccuracies or errors in the data or analysis that undermines the results, which can lead to corrections or retractions [46] and weaken trust in the scientist and science in general [47].

To reduce the insecurity associated with the public sharing of data and code, researchers can first share materials with trusted co-authors or peers in a safe environment, like laboratory meetings or code sharing clubs. Pre-print servers can also provide a lower-stakes venue for soliciting feedback on code and analyses prior to formal peer-review (e.g. *bioRxiv*). At the time of manuscript submission, data and code should be shared with the journal peer-reviewers to improve the quality of the manuscript and supporting materials [48–50]. Getting feedback on code and documentation at all of these stages can improve its efficiency, clarity and utility beyond an individual project. Many repositories (e.g. Open Science Framework and Dryad) allow for a private ‘peer-review’ data and code sharing link if authors do not wish to make their products available to the public until after the review process is complete. It is important that peer-reviewers assess the quality of the data and code products themselves and report on whether there are sufficient metadata to understand such products [51,52]. If authors do not submit data and code for peer-review, we suggest that reviewers and editors recommend authors upload such products before final acceptance. Many peer-reviewers may not have the expertise to review such products. If this is the case, we encourage peer-reviewers to explain this to journal editors, who would benefit by explicitly soliciting peer-review of the data and code itself. Once published, code usage will generate additional feedback that will improve functionality and fix errors. It is also important to recognize that there is no such thing as ‘perfect code’. There will always be trade-offs (e.g. among clarity, efficiency, ease and longevity) and there are diverse opinions about best practices for scientific software [53–56].

Furthermore, the process of cleaning and reviewing data and code for publication will usually reveal errors to the author before they are exposed publicly, which leads to higher-quality results than if data and code were not going to be published. If someone identifies a mistake in your data or code, this can easily be updated in the submission of a new ‘version’ (e.g. via Zenodo, Dryad or Figshare) of data and code. If this mistake changes the results of your published article, there is precedent for gracefully issuing a correction or, much more rarely, a retraction [46]. As a scientific community, we should continue to applaud those who acknowledge and correct human errors. By fostering a more inclusive, kind environment that emphasizes growth and learning over criticism and shame, we will reduce individual insecurities and fear associated with publicly sharing data and code [57,58].

### (e) Do not see the value

Researchers may not envision that anyone else would be interested in their data or code and, therefore, do not see the value in sharing it. This may be particularly common when there are low sample sizes, a limited scope of data collection (e.g. in terms of geography, taxonomy and time), large amounts of uncertainty or error, and/or relatively simple or straightforward scripts. A review by Perrier *et al*. [59] suggests that regardless of the reason why people place low value on data sharing, this value judgement is an inherently subjective rather than an objective decision.

Uncertainty about potential reuse should not present a barrier to sharing, as there is a multitude of ways that a given set of data or code could be used by future generations of scientists, which is one reason why major funding agencies and many journals are now requiring open data and code products. Advances in science and technology allow for data reuse that the original data collectors never could have imagined [9,60,61]. In other cases, a dataset of poor quality or limited sampling may represent the only set of data on a particular subject, and its rarity may increase its value despite its shortcomings, as in the case of data on endangered species [62]. Moreover, data are often useful in novel synthesis analyses that may explore research questions entirely unrelated to the original motivation of the data collection. The open science movement is value-driven in pursuit of improved science, and by sharing data and code, we might contribute to interdisciplinary knowledge integration [63]. For example, open collaboration is exemplified by the growth of open projects on public platforms such as GitHub, where collaborators can add value to existing code by integrating their own ideas and knowledge [64]. The more information we leave for future researchers, the better they will be able to progress our understanding of the world around us.

## 3. Reuse concerns

### (a) Inappropriate Use

Many scientists worry that, if they share their science openly, others will misinterpret their data or use their data and code inappropriately [1]. Those who are less familiar with the nuances of a particular data collection or analytical approach may overlook confounding factors and assumptions or draw erroneous and misleading conclusions through reuse.

Fortunately, researchers can take steps to reduce or avoid the inappropriate use of data and code. Data and code can be published alongside detailed metadata information, or with a data paper in an indexed, peer-reviewed journal, including a thorough description of datasets and processes, terms and considerations for reuse, and any limitations, assumptions, caveats, and shortcomings [65]. When one is accustomed to the nuances or assumptions of methods that they frequently use, it can be easy to forget to include important information that would allow others to replicate the study. Thus, metadata descriptions ideally would be looked over by someone other than the original researchers (e.g. peer-reviewers, friendly colleagues, etc.), who might more easily catch these omissions. Dryad data repository will review submitted metadata to some degree (while most free repositories do not), but this process could benefit from an explicit call to review metadata during journal peer-review.

Yet, open data that include thorough metadata can still omit important information that only the original data collectors had access to (e.g. idiosyncrasies of specific field sites or sampling years). Thus, researchers should also include contact information and an invitation for others to collaborate and/or reach out for assistance in interpreting and using the data and code. Being open to helping others reuse data and code is the best way to avoid misinterpretation, and it may also create opportunities for new collaborations in research areas the original researchers would have never thought to pursue. Yet, it can often be unclear as to who the primary contact for a dataset should be and it is worth considering the longevity of such information. Early-career researchers lead most research [66–68] (but see [69]), yet high turnover in positions [70–72] means that institutional emails frequently become outdated, and principal investigators may not have the capacity or knowledge to respond to inquiries. To ensure continuity in contact information, research groups may consider establishing a shared email address that can persist despite personnel turnover. At the institutional level, financial support from government and funding agencies can further help research programs to maintain continuity, for example by hiring database managers who ensure that data and code products and metadata are well documented and available for future use.

One may also opt to publish data and code in repositories that allow the contributor to set the permissions and rights of access and reuse. For example, certain licences will require acknowledgement, prevent data being used for commercial purposes or being modified without the permission of the owner (see §3b below). Researchers can contact authors or journals to solicit a correction when there is data misuse, or publish a response to ensure the community is aware of it, although this process can be lengthy and complex and may not ultimately change the scientific record or narrative. Lastly, it is important to note that all forms of scientific products can be misused [73,74]. One can just as easily cite previous work erroneously or misinterpret findings in an article's discussion and/or data presented in its figures and tables as one can in data and code shared with the public—this is no reason not to publish these scientific products.

### (b) Rights

Researchers may understandably feel a sense of ownership over data and code that they generate and may be hesitant to give up their exclusive right to use them. Furthermore, data and code may have complex ownership involving multiple people and institutions, complicating sharing efforts [75–77]. For example, research may have been conducted collaboratively, data may legally belong to an institution or funder rather than an individual researcher or data may be derived or synthesized from other primary datasets with different owners. Ownership of data and code may be further complicated after the publication of the original research article. Some publishers require a copyright assignment to the journal at the time of submission of a manuscript, which might include data and code products.

It is important to remember that we do not often have exclusive ownership of data to begin with. In cases where research is funded by federal government or public agencies (including, for example, the National Science Foundation and National Institutes of Health in the United States), researchers are obligated to publicly share research products that were generated through public funds for the benefit of society [11]. Institutional libraries and offices dedicated to copyright, open science and commercialization provide support and resources that can help researchers navigate the legal and ethical aspects of ownership and rights [29]. Data and code licences that define terms and conditions of reuse exist to protect researcher rights. In the context of collaborations, sharing agreements made early in the research process can specify the plans for ultimately sharing data, derived data products and code. When dealing with institutional or journal claims to research outputs, researchers should be aware of relevant policies and seek help clarifying the legal implications of institutional partnerships. When data and code are uploaded directly to a journal, those data and code products may be subjected to the same paywall as the article itself. Instead, considering the more general open repositories (listed in §2a above) can lead to increased accessibility and longevity (see §3d below) of data and code.

### (c) Sensitive content

There are some situations in which publicizing data may not serve the best interest of science and society, and it should instead remain private [78]. This is sometimes referred to as the ‘dual-use’ dilemma [79], originally coined to describe the potential for biological data to be usurped for the purpose of bioterrorism. Within biology, notable scenarios that invoke the dual-use dilemma are sharing the location data for species under threat of poaching, capture for the pet trade [80], significant harassment or disturbance to species or their habitat from their whereabouts being exposed [81], private information about individuals [82] or individual interviews that are not meant to be public [83–86].

Researchers, communities and institutions, where appropriate, should weigh the benefits and costs (to individuals, local communities and society at large) of publishing data. In some cases, aggregating, generalizing or anonymizing data can be used to remove sensitive information. In the context of biodiversity conservation [87], there are guidelines regarding the assessment of the sensitivity of the species and the choice of appropriate levels of generalization and masking (either of the species' identity or location) using resources such as those provided by the Global Biodiversity Information Facility (GBIF; [88]). Sharing detailed metadata with a limited subsample of the data can help inform other scientists or stakeholders of the existence and utility of the data you possess [89]. This public-facing data description can include reliable correspondence information and an invitation to request the data privately (although sharing data privately rather than publicly should be done sparingly) [90]. These incomplete datasets can also allow users to test the operation of the accompanying analysis code, without jeopardizing the sensitive information found within the data.

Additionally, generating synthetic data can be used to provide proof of concept without violating ethics of sharing sensitive data. Within the biomedical field, technical solutions have been developed for sharing synthetic data that capture the statistical properties of the original dataset, including sequential data synthesis using regression and classification trees [91] and software frameworks like statistical health information release (SHARE; [92]). These methods are being developed and generalized toward fields outside of biomedicine, including accessible resources like the *synthpop* R package for synthetic data generation [93].

Importantly, it is necessary to consider how individuals and communities will be impacted by the publishing of certain information. Sharing interview data, for example, without explicit consent from the interviewee is unethical, and reuse of this information out of context of the framing of the interview and questions can be problematic. In particular, many communities distrust science due to historic and ongoing harm, and special sensitivity is warranted in these cases. For example, Indigenous peoples and their data have been exploited and their natural resources abused by governments and commercial interests globally [83–86,94]. As we collectively move toward open data practices, we (as individuals, institutions, journals and funders) need to recognize the continued injustices to marginalized groups and advocate for data sovereignty. While open data is an important goal for advancing science, it must never perpetuate harm, and there are therefore circumstances in which data are best left unshared.

### (d) Transient storage

Researchers may be reluctant to spend time making data and code publicly available if they are unsure of the usability of such products over the long term. Data and code may not be available indefinitely, given the lack of infrastructure for long-term storage facilities, proprietary storage formats and evolving software. Short-term storage options, such as GitHub and cloud-based storage (e.g. Google Drive, OneDrive and Dropbox), offer no promise of permanency as accounts (and thus data and code) can be deleted at any time by the user. Similarly, promises such as ‘The raw data/analysis code supporting the conclusions of this article will be made available by the authors, without undue reservation’ cannot be fulfilled if those authors lose hard drives, change email accounts, leave academia, or are deceased [90,95].

Researchers should archive their data in repositories that have the greatest likelihood of permanent support and maintenance, which are rarely the journals themselves. Some long-term generic storage infrastructure, such as Dryad and Zenodo, assign digital object identifiers (DOIs) and will retain all files for the lifetime of the repository. Some organizations or academic journals cover costs of long-term data archiving (e.g. CERN with Zenodo and The Royal Society journals with Dryad, respectively), while some funding agencies provide funding for long-term storage costs to their grantees (e.g. Wellcome Trust [96] and NIH [97]). Ultimately, securing funding to ensure long-term storage and usability of code is a community-driven goal that will require research institutions, funders and publishers to work together [98,99].

Additionally, researchers should avoid proprietary file formats and software, such as Microsoft suite (e.g. .doc and .xlsx formats), SAS or SPSS data formats [100]. These products are subject to the stability and consistency of these programs (and any required packages and dependencies) and the continued support for older file formats. To the extent possible, researchers should use stable, non-proprietary file formats (e.g. comma separated value, .csv, for data and plain text files, .txt, for documentation, provenance and metadata files). Another benefit of providing source code is that it can still be examined visually to reproduce past work, even if the code no longer runs properly due to different running environments, versioning issues or a lack of continued availability of software dependencies [101,102].

Researchers can make use of tools that promote backwards compatibility and portability of software and packages within different operating systems. These tools include software containers, which store all packages used alongside the code [103] (e.g. Docker, originally designed for app developers, ‘renv’ for R [104] and ‘conda’ for Python (https://docs.conda.io/en/latest/)). Bindr (https://mybinder.org) allows users to interactively run code (e.g. R, Python, Julia, etc.) on Jupyter notebooks, which might be stored remotely on a GitHub repository (for example: https://github.com/geo-yrao/esip-ml-tutorials).

## 4. Disincentives

### (a) Scooping

One of the major barriers to data and code sharing is a fear of being ‘scooped’. Scooping in this context colloquially refers to a situation in which a researcher performs analyses on publicly shared data that the original data collector had planned, but not yet completed themselves [105,106]. Potential code sharers may also fear that freely sharing code will reduce opportunities for collaboration and co-authorship with other researchers who may be interested in using their code. Furthermore, long-term datasets may not result in papers immediately, and researchers may be concerned that releasing data too early may compromise their ability to publish. The potential loss of future publications represents a cost in the context of today's scientific landscape, where publications are a key metric used to assess research productivity amidst competition for grants and positions [78,107].

Getting scooped is less likely than one might imagine, given that ideas are plentiful and diverse, and that those who collect data and develop code remain best positioned to undertake future analyses [108,109]. Researchers publish most papers using their own datasets within 2 years of original publication, while papers that cite open datasets peak at 5 years after data publication [18]. Additionally, pre-print servers offer the ability to make first claim to a research project through rapid dissemination of one's work and ideas [110]. Sharing how one collected open datasets along with any preliminary analyses or visualizations can alleviate concerns of being scooped when researchers do not immediately have time to go through the entire peer-review process. In these cases, pre-printed articles are already citable (with a DOI) and benefit from increased viewership, citation rates and collaborations [110–112] (but see [113] for concerns regarding pre-printing sensitive information, and §3c above).

If scooping is a major concern, there are ways to communicate expectations about how data should be used (e.g. see §3d above). In general, however, individual careers and scientific progress are advanced when we take a cooperative, collaborative approach [18,57,114], and data sharing will increase, rather than decrease, opportunities for collaboration. Institutions and funding agencies can alleviate scooping concerns and promote open science practices by viewing shared datasets and code as products that can be, in themselves, just as valuable as publications (see §4c below). Researchers who have spent their time, energy and finances on long-term datasets should receive appropriate credit (e.g. via promotion and future funding opportunities) for collecting such important data, regardless of whether these same researchers have led any scientific publications using the datasets. Giving disproportionate credit to new analyses, rather than new data collection efforts is limiting our knowledge and collective willingness to be open with our work. As a community, we should be more inclusive of those who generate the data we use in our research. Those who have collected data are instrumental to a research project, and their participation in the development of a publication should be thoughtfully considered. At a minimum, care should be taken to follow the appropriate permissions and rights of access and reuse, and data should be properly cited (see §3 above).

### (b) Lack of time

Researchers may be reluctant to share their data and code because of the perceived short- and long-term time commitments required to do so [21]. In the short term, it can take significant time to clean, prepare and annotate data, code and metadata for archiving, especially if these were not well organized from the beginning of the research project (for some guidance on that, see §2a above). In the long term, researchers may be reluctant to commit to ongoing curatorial support of others who try to reuse their data or code (see §4c below).

Despite the upfront time required, sharing research data and code can ultimately save time for individual researchers and their collaborators, as well as for others who want to reuse it. A researcher's most important collaborator is their future self [57], and the practice of annotating and organizing data and code is ultimately most useful to oneself. For example, archiving data in a long-term repository (e.g. Dryad, Figshare and Zenodo) ensures that users always have access to their own data and code files regardless of switching institutions or computers. Beginning a research project with the understanding that data and code will eventually be shared can generally lead to better standards, workflows and documentation throughout, and can reduce the time required for editing and cleaning once the project is complete [35]. The preparation of data and code should be considered as important as other publication tasks like managing citations and editing manuscript grammar, and should be prioritized in project management and delegation of roles within a team [90,98]. Research institutions can support this work by hiring designated data management teams that work with individual researchers, likely housed within institutional libraries [29]. Finally, the creation of supporting documents like descriptive metadata and readme files that include data and code version information [56] can help to ensure that the files are reusable in the long-term without further time commitment from the researcher.

### (c) Lack of incentives

In addition to all of the perceived costs of sharing data and code outlined above, there is also a lack of perceived benefit among many researchers [59]. There have historically been few apparent career incentives to making one's data and code publicly available [115]. However, as discussed in the sections above, there are actually more career benefits to sharing data and code than one might realize.

Sharing data and code can increase visibility and recognition of a researcher within the scientific community, which may initiate new collaborations between data sharers and data reusers [116]. It can also help develop open science habits that increase efficiency, and contribute to a better understanding of one's own data and code (e.g. by providing descriptive metadata for files or commenting code). Research papers that include an access link to the primary data are cited significantly more often (25–69% more often) than papers that do not provide access to their data [18,20,117–119]. Data and software journals more frequently publish data and code with their own DOI, which allows data and code to be persistent, searchable, findable and formally cited. Thus, data and code uploads can be cited themselves, or included in a more comprehensive, stand-alone data paper (see https://www.gbif.org/data-papers) that is also citable—and at times to a high degree (https://www.earth-system-science-data.net/).

Increasingly, data and code sharing are being incentivized or even required by funders and publishers of scientific research [95,120,121]. Over the past decade, funding institutions have been acknowledging the importance of public data sharing in accelerating scientific discovery and advancement. Many recent recommendations for public funding agencies require that data and software generated with public funds be provided freely (e.g. OECD Council (https://www.oecd.org/); the U.S. White House [11]), and that funders should consider the value and impact of all research outputs (including data and software) in addition to publications (e.g. San Francisco Declaration on Research Assessment (DORA) (https://sfdora.org/read/); [122]). It is now common for funding institutions to require data sharing statements or data management plans in grant proposals, and to use those as part of funding allocation decisions. In the United States, the National Institutes of Health (NIH) has required data sharing since 2003 [97,123] and the National Science Foundation (NSF) [124] has required a data management plan for grant proposals since 2011. The NSF explicitly expects grantees to share primary data, and failure to comply with data management plans may negatively influence future funding opportunities, or result in the withholding or adjustment of funds [125]. Similarly, many scientific journals now require or strongly encourage data and code to be published alongside manuscripts [126]. The policing of such policies, however, could use strengthening.

Employers and academic institutions have been slower to incentivize data and code sharing with either rewards or punishments, but some institutions are beginning to value these practices among their employees. For example, in 2021, NASA launched their ‘Transform to Open Science’ initiative in which they proposed a number of incentives to reward and recognize data sharing actions. As part of this initiative, they are establishing an Open Source Science Award Program and aiming to incorporate open science activities into their reviews system. Professional societies are also granting awards to practitioners of open science, including SORTEE.

We hope that as more researchers recognize the value of open science, the publication of data and code will be considered in hiring, tenure and promotion [127]. Indeed, we are not alone in this desire, as the DORA begins: ‘There is a pressing need to improve the ways in which the output of scientific research is evaluated by funding agencies, academic institutions and other parties' [128]. As of 31 August 2022, 22 081 individuals and organizations across 159 countries have signed the declaration. DORA outlines the importance of data and software products in individual outputs and makes specific recommendations for funding agencies, institutions, publishers and individual researchers (https://sfdora.org/read/).

## 5. Conclusion

We recognize that there are many real and perceived costs and barriers to sharing scientific data and code (figure 1). In many cases, on an individual level, perceived barriers may be relatively easily overcome (e.g. lack of knowledge) or may not actually present insurmountable obstacles (e.g. large file sizes). In other cases, the associated downstream benefits to research efficiency, productivity and collaboration may ultimately outweigh costs (e.g. time investment, fear of scooping). It is our hope that by outlining the above barriers to data and code sharing, we have enabled researchers to reflect on their own experiences and practices in order to recognize and mitigate the most salient barriers that they face.

As individuals, we should all make an effort to share well-documented data and code with clear and open lines of communication, which will reduce risks of data misuse while advancing the scientific enterprise. As members of our scientific communities, we should foster a culture that celebrates open science practices by our peers and advocate for incentives to share data and code in the context of research funding, publication and career evaluation. That is, data and code products are useful contributions to science on their own and should be valued as such. As journal editors and reviewers, for example, we can request that authors include data and code with their papers for peer-review—whether or not we have the skills or time to also review those products. Yet, we should be open about this lack of knowledge and journal editors (and authors) should explicitly solicit review of data and code products. Open science has great potential to advance the pace of scientific discovery while fostering a more collaborative and cooperative research environment, and publicly sharing data and code is a critical step towards these goals.

## 6. Process and authors' contributions

The initial discussion took two hours, was open to any who wanted to join, and was freely available via the SORTEE organisation and conference programme. In the discussion, we collaboratively brainstormed barriers to open data and code, drawing first from our own experiences as individual researchers. All SORTEE conference participants were invited to follow up to write this paper distilling our initial discussion about why we think individuals are reluctant to share data and code and to refine some counter points to these arguments. Those who had opted in participated in three follow-up discussions focused on consolidating and fleshing out the final list of barriers based on our experiences as well as the published literature. We all collaboratively compiled information and references for these arguments and counter-arguments. Each of us then drafted an individual section, followed by group edits. D.G.E.G., R.C.-O. and K.M.G. made final edits for consistency and clarity and P.P. made the central figure with feedback from all authors.

## Data Availability

The supplementary notes are provided in the electronic supplementary material [129].
